# Coordinated innate and T-cell immune responses in mild COVID-19 patients from household contacts of COVID-19 cases during the first pandemic wave

**DOI:** 10.3389/fimmu.2022.920227

**Published:** 2022-07-27

**Authors:** Alessandra Aiello, Adriano Grossi, Silvia Meschi, Marcello Meledandri, Valentina Vanini, Linda Petrone, Rita Casetti, Gilda Cuzzi, Andrea Salmi, Anna Maria Altera, Luca Pierelli, Gina Gualano, Tommaso Ascoli Bartoli, Concetta Castilletti, Chiara Agrati, Enrico Girardi, Fabrizio Palmieri, Emanuele Nicastri, Enrico Di Rosa, Delia Goletti

**Affiliations:** ^1^ Translational Research Unit, National Institute for Infectious Diseases Lazzaro Spallanzani-IRCCS, Rome, Italy; ^2^ Local Public Health Office, Azienda Sanitaria Locale (ASL) Roma 1, Rome, Italy; ^3^ Laboratory of Virology, National Institute for Infectious Diseases Lazzaro Spallanzani-IRCCS, Rome, Italy; ^4^ Unità Operativa Complessa (UOC) Microbiology and Virology, Azienda Sanitaria Locale (ASL) Roma 1-San Filippo Neri Hospital, Rome, Italy; ^5^ Unità Operativa Semplice (UOS) Professioni Sanitarie Tecniche, National Institute for Infectious Diseases Lazzaro Spallanzani-IRCCS, Rome, Italy; ^6^ Laboratory of Cellular Immunology, National Institute for Infectious Diseases Lazzaro Spallanzani-IRCCS, Rome, Italy; ^7^ Unità Operativa Complessa (UOC) Transfusion Medicine and Stem Cell, San Camillo Forlanini Hospital, Rome, Italy; ^8^ Respiratory Infectious Diseases Unit, National Institute for Infectious Diseases Lazzaro Spallanzani-IRCCS, Rome, Italy; ^9^ Clinical Division of Infectious Diseases, National Institute for Infectious Diseases Lazzaro Spallanzani-IRCCS, Rome, Italy; ^10^ Clinical Epidemiology, National Institute for Infectious Diseases Lazzaro Spallanzani-IRCCS, Rome, Italy

**Keywords:** household contacts, COVID-19, SARS-CoV-2, T-cell response, Interferon-alpha (IFN-α), Interferon-gamma (IFN-γ) release assay (IGRA), whole blood, spike protein

## Abstract

**Objective:**

To better define the immunopathogenesis of COVID-19, the present study aims to characterize the early immune responses to SARS-CoV-2 infection in household contacts of COVID-19 cases. In particular, innate, T- and B-cell specific responses were evaluated over time.

**Methods:**

Household contacts of COVID-19 cases screened for SARS−CoV−2 infection by nasopharyngeal swab for surveillance purposes were enrolled (T0, n=42). Of these, 28 subjects returned for a follow-up test (T1). The innate response was assessed by detecting a panel of soluble factors by multiplex-technology in plasma samples. Cell-mediated response was evaluated by measuring interferon (IFN)-γ levels by ELISA in plasma harvested from whole-blood stimulated with SARS−CoV−2 peptide pools, including spike (S), nucleocapsid (N) and membrane (M) proteins. The serological response was assessed by quantifying anti-Receptor-Binding-Domain (RBD), anti-Nucleocapsid (N), whole virus indirect immunofluorescence, and neutralizing antibodies.

**Results:**

At T0, higher levels of plasmatic IFN-α, IL-1ra, MCP-1 and IP-10, and lower levels of IL-1β, IL-9, MIP-1β and RANTES were observed in subjects with positive swab compared to individuals with a negative one (p<0.05). Plasmatic IFN-α was the only cytokine detectable in subjects with positive SARS-CoV-2 swabs with high accuracy for swab score positivity (0.93, p<0.0001). Among subjects with positive swabs, significant negative correlations were found among the RT-PCR cycle threshold values reported for genes S and N and IFN-α or IP-10 levels. At T0, the IFN-γ T-cell specific response was detected in 50% (5/10) of subjects with positive swab, while anti-RBD/anti-N antibodies showed a positivity rate of 10% (1/10).

At T1, the IFN-γ T-cell specific response was detected in most of the confirmed-infection subjects (77.8%, 7/9), whereas the serological response was still observed in a minority of them (44.4%, 4/9). Overall, the swab test showed a moderate concordance with the T-cell response (78.6%, k=0.467), and a scarce concordance with the serological one (72.9%, k=0.194).

**Conclusions:**

Plasmatic IFN-α and the IFN-γ T-cell specific response appear early even in the absence of seroconversion, and show a greater positivity rate than the serological response in household contacts with positive swab.

## Introduction

The COronaVIrus Disease (COVID-19) caused by the Severe Acute Respiratory Syndrome Coronavirus 2 (SARS-CoV-2) is a new zoonosis that has spread since December 2019. SARS-CoV-2 infection occurs with a variety of clinical syndromes; most people present a less severe disease and remain asymptomatic or paucisymptomatic, while approximately 20% of people (at least with the ancestral strain) develop severe respiratory symptoms, which may lead to hospital admission and eventually to death (1–4). The host itself seems to be the major factor explaining disease severity, infection rates (5, 6), and long-term medical consequences (7).

The host response to SARS-CoV-2 infection described during the first epidemic wave, before vaccination, is crucial to understand the mechanisms of effective host-defence in naïve populations. Evidence indicates that a coordinated innate and adaptive immune response, that includes both T and B cells, is necessary to mount an appropriate immune protection that counteracts SARS-CoV-2 infection (8, 9). The T-cell response is also crucial against the variants of concerns (10).

Soluble factors including cytokines, chemokines and growth factors act, both locally and systemically, influencing the release of innate immune cells from the bone marrow into circulation, as well as their recruitment to inflamed and infected tissues (11).

The variability in innate immune system components has been correlated to the heterogeneous disease courses observed in COVID-19 patients (12). Increased pro-inflammatory or anti-inflammatory cytokines, including T helper type-1 and type-2 cytokines and chemokines, were reported (13–16). Interleukin (IL)-1β, IL-6, IL-8, and Interferon (IFN)-γ-inducible protein (IP-10) were found to be correlated with severe or fatal outcome (17–19). Strong evidence has also shown that innate immunity mediated by type I IFN responses contributes to protection against critical illness (20–22).

Many studies evaluating immune correlates of protection against SARS-CoV-2 focused on the detection of neutralizing antibodies (23–26). However, antibodies are absent in the early stages of the disease or not detectable in patients with less severe forms of COVID-19 (27–29). Levels of neutralizing antibodies are highly variable (1) and antibody titers wane over time (30, 31). Moreover, there is no agreement on the cut off and, so far, no correlates of protection are available.

Conversely, T-cell response is detectable during acute disease and in recovery and is more durable (32–35). Different works validated a whole-blood test based on IFN-γ release for the detection of a SARS-CoV-2 specific T-cell response to discriminate COVID-19 patients from uninfected individuals, and to monitor the immune response in vaccinated individuals (13, 36–40).

Both innate and adaptive immune responses are involved in virus clearance, inhibition of virus replication and promotion of tissue repair. Lack of coordination among the immune responses has been associated with poor outcome as in the elderly (41). Accordingly, it is of great importance to evaluate the combination, as well as the timing (kinetics) of both immune responses against COVID-19 disease, starting from the earliest stages. This acquired knowledge can be useful for new diagnostic tools or therapy interventions. However, there is a lack of longitudinal studies on the combined analysis of innate immunity, serological and T-cell specific responses against SARS-CoV-2 in the same patient population during the first epidemic wave (42). Therefore, in this study, we characterized the innate and adaptive immune responses in individuals early exposed to SARS-CoV-2, who have presented an asymptomatic or mild COVID-19, correlating the results with the outcome of the nasopharyngeal swab.

## Materials and methods

### Study population

The prospective study was conducted between February 10^th^ and June 17^th^ 2021. The Ethical Committee of the National Institute for Infectious Diseases (INMI) L. Spallanzani approved the study (approval number 247/2021).

The study population was selected based on the probability of having or not a recent SARS-CoV-2 infection compared to a control population. Household contacts of confirmed COVID-19 cases were enrolled among the subjects screened for surveillance purposes at the drive in at the ASL Roma 1 Santa Maria della Pietà (Rome, Italy). These subjects were screened for SARS-CoV-2 infection by nasopharyngeal swab to detect early household’s positivity. The enrolled subjects were tested at two time points: at the execution of the first swab (T0) and for a follow-up test after 7-20 days (T1), end of the quarantine period.

In addition to these individuals, 53 COVID-19 patients with acute disease were recruited as positive controls. Inclusion criteria for COVID-19 patients were a diagnosis based on a positive nasopharyngeal swab for SARS-CoV-2 and a disease with the clinical characteristics already described (43). The COVID-19 group included patients with a moderate, severe or critical disease according to WHO (44). These patients were classified based on the highest severity score of the disease occurring during the hospitalization as described (36, 45).

“NO-COVID-19”-individuals were enrolled among healthy blood donors (HD) from Transfusional Medicine and Stem Cells Unit at the San Camillo Forlanini hospital (Rome, Italy). Inclusion criteria for the “NO-COVID-19” group were: negative SARS-CoV-2 serology and/or negative swab and no symptoms of COVID-19.

Exclusion criteria for the enrollment were: HIV infection, having received vaccination against SARS-CoV-2, communication of a previous SARS-CoV-2 infection, incapability to sign an informed consent, and age ≤18 years.

All the enrolled patients and controls signed a written informed consent. Demographic and clinical information were obtained at the time of enrollment.

### SARS-CoV-2 molecular testing

The molecular research of SARS-CoV-2 in nasopharyngeal swabs was performed using Seegene automated instrumentation (Seegene Inc., Seoul, Republic of Korea). The procedure involves the extraction of RNA using the NIMBUS/STARlet system and STARMag Universal Cartridge kit. The instrumentation automatically sets microplates, which are then processed on the real-time PCR Biorad CFX96 (Bio-Rad Laboratories, CA, USA). Seegene’s Allplex SARS-CoV-2 Assay was used as real-time PCR method; it detects, in a single tube, RdRP, S and N genes for SARS-CoV-2. Allplex SARS-CoV-2 Assay was the evolution of the technology developed by Seegene in 2020, based on the Corman protocol (46).

### SARS−CoV−2 peptide pools

Stimulations were performed with peptide pools of 15 amino acid length with an 11 amino acid overlap encompassing the sequence of the SARS−CoV−2 spike protein (PepTivator^®^ SARS-CoV-2 Prot_S1, Prot_S, and Prot_S+), nucleocapsid phosphoprotein (PepTivator^®^ SARS-CoV-2 Prot_N), and membrane glycoprotein (PepTivator^®^ SARS-CoV-2 Prot_M). A final concentration of 0.1 µg/mL was used for S and M peptide pools, whereas a concentration of 1 µg/mL was used for N peptide pool according to a previous study (36). All peptides were purchased from Miltenyi Biotec (Bergisch Gladbach, Germany).

### IFN-γ whole-blood assay

Cell-mediated immune response was evaluated using an IFN-γ release test based on the stimulation of whole-blood. Briefly, heparinized whole-blood (600 µL) was stimulated or not with SARS−CoV−2 peptide pools in a 48-well flat-bottom plate and incubated at 37°C (5% CO_2_) for 20-24h. After overnight stimulation, plasma was harvested and stored at -80°C until further analysis. IFN-γ levels were quantified by ELISA, according to manufacturer’s instructions (www.quantiFERON.com) and reported after subtracting the unstimulated control. The detection limit of the test was 0.065 IU/mL. For pools S and N, IFN- γ levels ≥ 0.13 IU/mL indicated a positive response, whereas for pool M the cut-off was ≥ 0.19 IU/mL according to the receiver operating characteristic (ROC) analysis performed comparing COVID-19 patients and “NO-COVID-19” subjects (36).

### SARS-CoV-2 antibody testing

The serological response was evaluated by measuring anti-Receptor Binding Domain (RBD) (ARCHITECT SARS-CoV-2 IgG II Quantitative, Abbott Laboratories, Wiesbaden, Germany), anti-Nucleocapsid (N) (ARCHITECT SARS-CoV-2 IgG, Abbott Laboratories) and neutralizing antibodies. Anti-N IgG are expressed as index values, i.e., Sample/Cutoff (S/CO), and values ≥ 1.4 indicate positive samples. Anti-RBD IgG are expressed as Binding Antibody Units (BAU/mL) and values ≥ 7.1 are considered positive.

The micro-neutralization assay (MNA) was performed as previously described (47), using the SARS-CoV-2/Human/ITA/PAVIA10734/2020 (isolated in March and provided by Fausto Baldanti, Pavia, Italy). The test is based on the evaluation of the cytopathic effect (CPE) at 48h after the infection of Vero E6 cells with 7 two-fold serial dilutions of the virus-serum mixture. The neutralization titer was expressed as the reciprocal of serum dilution (MNA_90_), i.e., the highest serum dilution inhibiting at least 90% of the CPE. The positivity threshold was set at 1:10.

### Indirect immunofluorescence assay (IFA)

To verify the specificity of cell-mediated and serological responses in subjects with negative swab, an indirect immunofluorescence assay was performed using home-made slides prepared with SARS-CoV-2-infected Vero E6 cells, as described elsewhere (48).

### Cytokines/chemokines evaluation

To evaluate the cytokine/chemokine profile, blood was collected in heparinized tubes and processed within 2 hours from collection. Briefly, plasma was separated by centrifuging the blood at 500 x g for 10 minutes, aliquoted and stored at -80°C until use. Unstimulated plasma samples were analysed using the Bio-Plex Pro Human Cytokine 27-plex Assay and the MagPix system, according to manufacturer’s instructions (all from Bio-Rad, Hercules, CA, USA). The multiplex allowed the detection of the following cytokines, chemokines and growth factors: interleukin (IL)-1β, IL-1RA, IL-2, IL-4, IL-5, IL-6, IL-7, IL-8, IL-9, IL-10, IL-12p70, IL-13, IL-15, IL-17A, eotaxin, basic fibroblast growth factor (FGF), granulocyte-colony stimulating factor (G-CSF), granulocyte-macrophage colony-stimulating factor (GM-CSF), IFN-γ, IP-10, monocyte chemoattractant protein-1 (MCP-1), macrophage inflammatory protein (MIP)-1α, MIP-1β, platelet-derived growth factor (PDGF), RANTES (regulated on activation, normal T-cell expressed and secreted), tumour necrosis factor-alpha (TNF-α), vascular endothelial growth factor (VEGF). Data were generated using the Bio-Plex Manager software. Concentrations below the detection range were considered zero and samples acquired with a bead count <50 were excluded from the analysis.

In addition, unstimulated plasma samples were tested for IFN-α and-β by an automatic ELISA (ELLA, protein simple, R&D System, Minneapolis, MN, USA). The limit of detection of IFN-α was 0.51 pg/mL, whereas for IFN-β was 1.03 pg/mL.

### Statistical analysis

The Graph Pad software (GraphPad Prism 8 XML ProjecT, San Diego, CA, USA) was used for the statistical analysis. IFN-γ levels and anti-RBD, anti-N and neutralizing antibody titers were reported as median and interquartile range (IQR), whereas categorical variables were reported as count and proportion. The following non-parametric statistical inference tests were used: Chi-square or Fisher’s exact test for categorical variables with Bonferroni correction when appropriate, Mann-Whitney U-test and Wilcoxon signed rank test for pairwise comparisons (for unpaired and paired data, respectively), the Kruskal-Wallis test and the Dunn’s multiple comparisons test for comparisons among groups. Correlations between assays were assessed by non-parametric Spearman’s Rank test. Spearman’s r_ho_ >0.7 was considered high correlation, 0.7 >r_ho_>0.5 moderate correlation and r_ho_<0.5 low correlation. ROC analysis was used for evaluating the area under the curve (AUC) and the diagnostic performance. Cohen’s kappa was used to assess the agreement between two assays. Two-tailed p-values <0.05 were considered significant.

## Results

### Description of the studied population

The study cohort consisted of 111 individuals. In particular, we prospectively enrolled 42 household contacts of laboratory-confirmed COVID-19 cases, and 53 COVID-19 patients and 16 “NO-COVID-19” individuals as control groups (**Table 1**).

**Table 1:** Demographical and clinical characteristics of the 111 enrolled subjects.

Characteristics		Household contacts	COVID-19 hospitalized patients	NO-COVID-19 subjects (blood donors)	P value
**N (%)**		42 (37.8)	53 (47.7)	16 (14.4)	
**Age median (IQR)**		48 (29-55)	58 (52-71)	42 (38-54)	<0.0001*
**Male N (%)**		20 (47.6)	34 (64.1)	12 (75.0)	0.104^§^
**Origin N (%)**	**West Europe**	39 (92.8)	50 (94.3)	16 (100)	0.434^§^
	**East Europe**	–	2 (3.8)	–	
	**Asia**	1 (2.4)	1 (1.9)	–	
	**South America**	2 (4.8)	–	–	
**Swab positive results at the time of enrolment N (%)**		10 (23.8)	53 (100)	0 (0)	
**Days from symptom onset N (%)**	**1-7**	–	22 (41.5)	–	
	**8-14**	–	21 (39.6)	–	
	**15-30**	–	8 (15.1)	–	
	**> 30**	–	2 (3.8)	–	
**Cycle threshold (Ct) values**	**gene S**	24.2 (19.6-32.3)	–	–	
	**gene N**	22.9 (19.2-31.1)	–	–	
**Days of exposure median (IQR)**		4 (4-5)	–	–	
**Time of follow-up N (%)**	**Available**	28 (66.7)			
	**≤ 7** ^a^	13 (46.5)			
	**8-14**	9 (32.1)			
	**15-20**	6 (21.4)			
**Severity N (%)^#^**	**Moderate**	–	14 (26.4)	–	
	**Severe**	–	30 (56.6)	–	
	**Critical**	–	9 (17.0)	–	
**Cortisone therapy N (%)**	**Available**	–	40 (75.5)	–	
			26 (65)		
**Other diseases** ^b^	**Available**	7 (16.7)	0 (0)	0 (0)	
	**Metabolic disease**	1 (14.3)	–	–	
	**Cardiovascular disease**	3 (42.8)	–	–	
	**Cancer in therapy**	1 (14.3)	–	–	
	**Thyroid disease**	2 (28.6)	–	–	
	**Neurological disease**	1 (14.3)	–	–	

COVID-19, coronavirus disease 19; N, number. *Kruskal–Wallis statistic test. ^§^Chi-square test. ^#^WHO criteria (ref WHO). The information regarding the hospitalized COVID-19 patients receiving or not cortisone was available only for 40 subjects (75.5%). Among these 40 subjects, only 26 (65%) were under cortisone therapy at the time of enrolment.

a Only one subject returned after 6 days.

b Of them, 2 subjects scored positive for the swab but they were able to mount both T-cell and antibody response.

**Figure 1:**
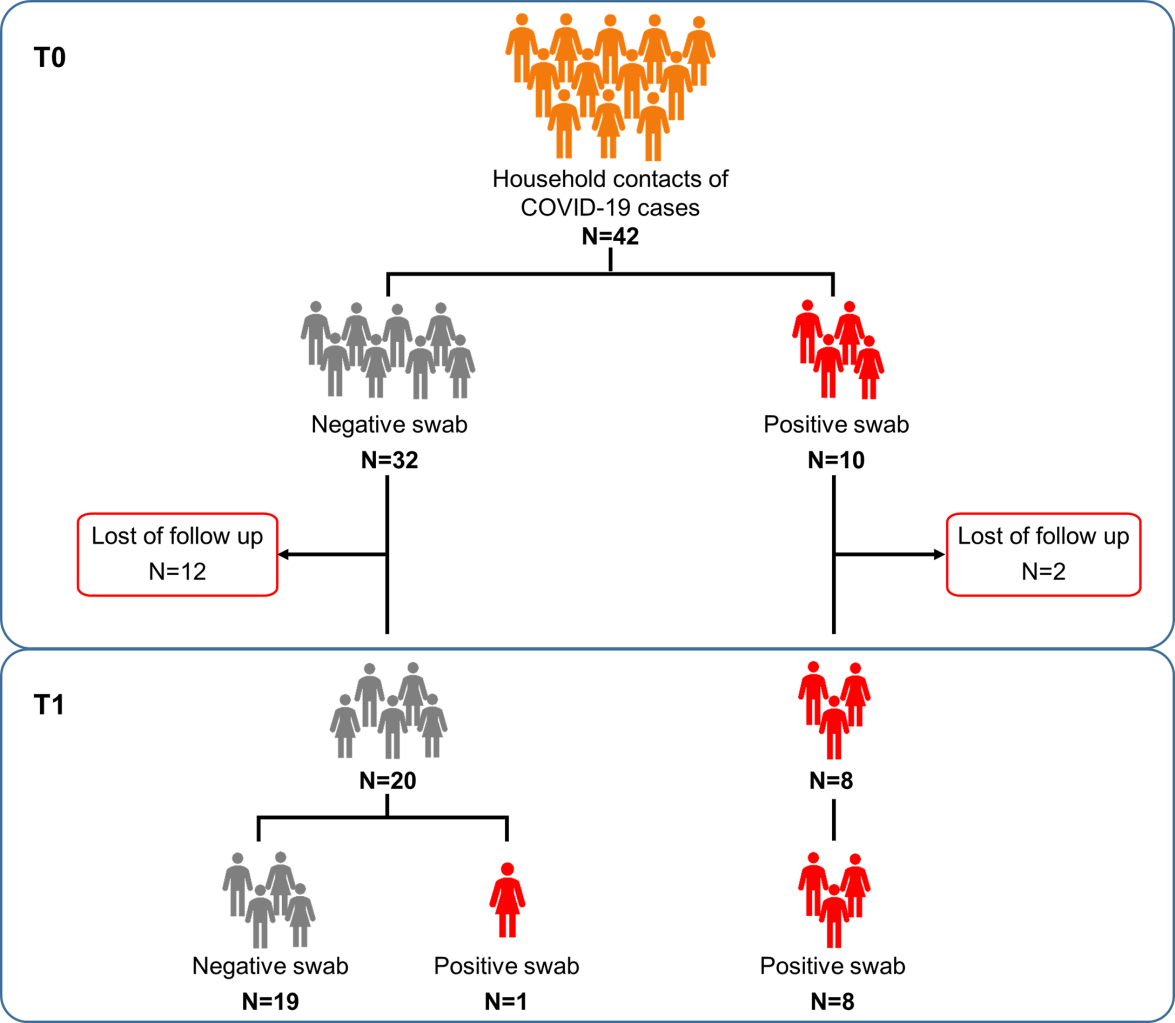
Flow chart of the enrolled household contacts of COVID-19 cases. Household contacts of COVID-19 cases (n=42) were enrolled and analyzed at the execution of the first nasopharyngeal swab (T0) and after 7-20 days (T1), at the end of the quarantine period. Twenty-eight of these subjects returned to follow-up. Footnote: COVID-19, COronaVIrus Disease 2019.

The three groups showed significant difference with respect to age (p>0.0001). Among household contacts, 10/42 (23.8%) scored positive for the swab on the day of sample collection (T0) (**Figure 1**). After 7-20 days (T1) from the first swab, 28 subjects returned to the follow-up. Among them, 19/20 of the subjects remained swab negative, whereas one individual, who scored negative in the first swab, became positive in the second one. All subjects scored swab positive at baseline remained positive at the follow-up (n=8) and had a mild COVID-19.

### A specific plasma cytokine/chemokine profile was found in household contacts with positive swab compared to those with a negative swab

**Figure 2:**
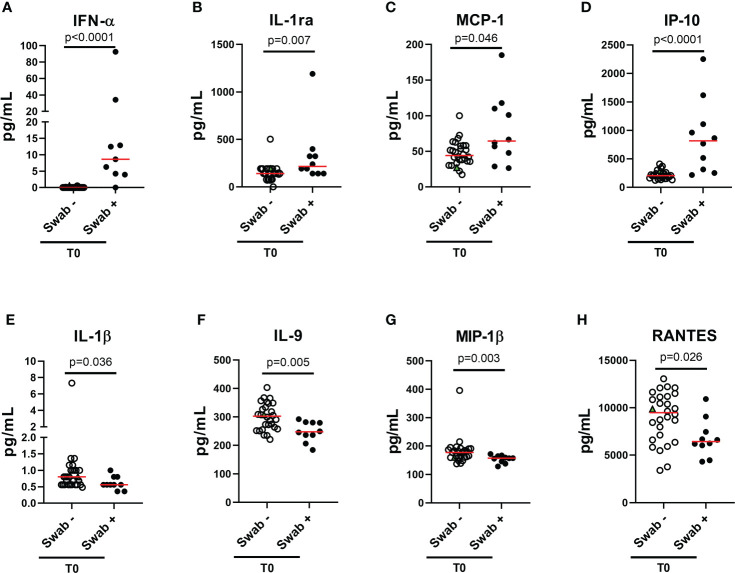
Plasmatic cytokines/chemokines modulated in household contacts at baseline. **(A–H)** Household contacts at T0 (n = 38) were stratified according to the swab result: positive (n = 10) and negative (n = 28). Plasma harvested from unstimulated blood samples were tested for the detection of 27 cytokines/chemokines using the Bio-Plex Pro Human Cytokine 27-plex Assay and for the detection of IFN-α and-β by means of an automatic ELISA. Red horizontal lines indicate medians. The green triangle identifies the subject with a positive swab only at T1. Statistical analysis was performed using Mann-Whitney U test to compare swab positive and negative subjects. A p < 0.05 was considered significant. IL, interleukin; MCP, monocyte chemoattractant protein; MIP, macrophage inflammatory protein; IP, Interferon-gamma induced protein; RANTES, regulated on activation IFN, interferon.

To characterize the cytokine/chemokine profile of early SARS-CoV-2 infections, the levels of several cytokines, chemokines and growth factors were assessed in the plasma of the enrolled household contacts at baseline (T0) and at the follow-up (T1). At T0, a different cytokine/chemokine profile was found in subjects with a swab positive result. In particular, significant higher levels of IFN-α, IL-1ra, MCP-1 and IP-10 were detected in the plasma of swab positive subjects compared to individuals with negative ones (p<0.0001, p=0.007, p=0.046 and p<0.0001, respectively) (**Figures 2A–D**). By contrast, lower levels of IL-1β, IL-9, MIP-1β and RANTES were found compared to swab negative subjects (p=0.036, p=0.005, p=0.003 and p=0.026) (**Figures 2E–H**). No significant differences were observed at the follow-up for these cytokines (**Supplementary Figure S1**) neither at both time points for the other soluble factors tested (**Supplementary Figures S2, S3**). ROC curve analysis showed that the highest AUC was associated with IFN-α (AUC: 0.93, 95% CI: 0.81-1.00, p<0.0001) followed by IP-10 (AUC: 0.92, 95% CI: 0.82-1.00, p<0.0001) (**Table 2**).

**Table 2:** Accuracy of the eight plasmatic cytokines/chemokines significantly modulated between swab-positive and swab-negative household contacts.

Cytokines/Chemokines	ROC AUC	95% CI	p
IFN-α	0.93	0.81-1.00	<0.0001
IL-1β	0.73	0.55-0.90	0.033
IL-1ra	0.77	0.60-0.95	0.011
IL-9	0.76	0.61-0.92	0.013
IP-10	0.92	0.82-1.00	<0.0001
MCP-1	0.70	0.48-0.93	0.057
MIP-1β	0.78	0.64-0.93	0.007
RANTES	0.71	0.54-0.88	0.048

AUC, area under the curve; CI, confidence interval.

Moreover, within the cohort of swab positive subjects, significant negative correlations were found between the RT-PCR Cycle threshold (Ct) values reported for genes S and N and IFN-α (IFN-α vs gene S: rho= -0.635, p=0.009 and IFN-α vs gene N: rho= -0.591, p=0.022), or IP-10 levels (IP-10 vs gene S: rho= -0.677, p=0.004 and IP-10 vs gene N: rho= -0.629, p=0.010) (**Table 3** **and** **Supplementary Figure S4**).

**Table 3:** Correlations between plasmatic cytokines/chemokines and RT-PCR cycle threshold (CT) values in swab-positive subjects.

Cytokines/Chemokines	CT gene S		CT gene N	
	rho	p	rho	p
IFN-α	**-0.635**	**0.009**	**-0.591**	**0.022**
IL-1β	-0.069	0.790	0.138	0.607
IL-1ra	-0.476	0.055	-0.376	0.151
IL-9	0.024	0.926	0.143	0.595
IP-10	**-0.677**	**0.004**	**-0.629**	**0.010**
MCP-1	-0.374	0.139	-0.302	0.254
MIP-1β	0.095	0.713	0.326	0.215
RANTES	-0.212	0.411	-0.072	0.790

S, spike; N, nucleoprotein. In bold are indicated the significant correlations.

In addition, no significant modulations were observed comparing the two time points, except for the chemokine MIP-1α that showed a significant increase at T1 in swab negative subjects (p=0.008) (**Supplementary Table S1**).

Interestingly, at T0 the IFN-α was the only cytokine specifically detectable in subjects with positive swab and no longer detectable in most of the subjects at follow-up.

### The IFN-γ-specific T-cell response to SARS-CoV-2 peptide pools was early detected in household contacts

**Figure 3:**
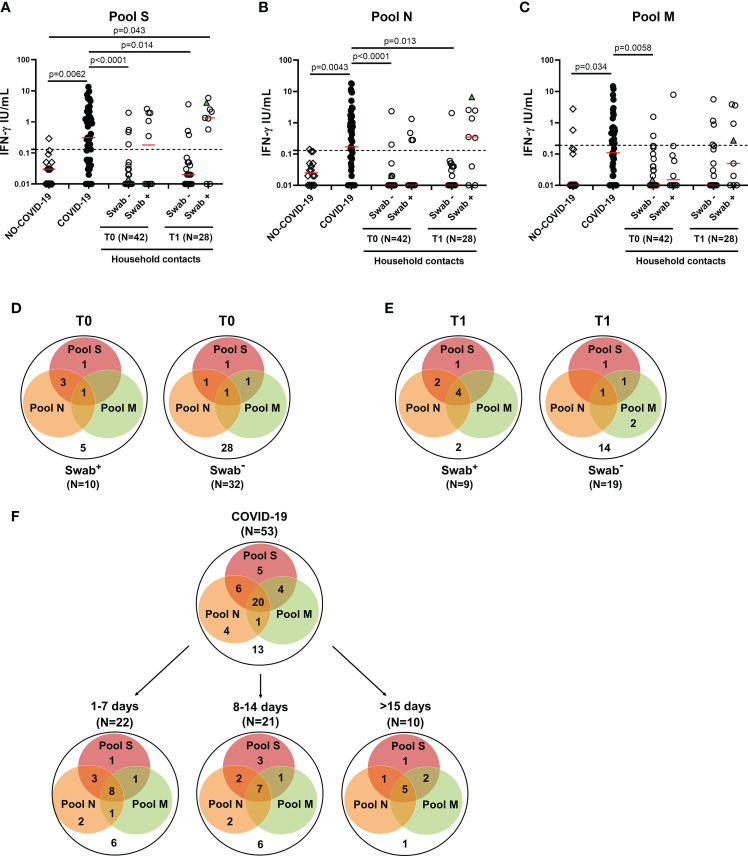
T-cell response in household contacts of COVID-19 subjects. **(A-C)** Evaluation of IFN-γ levels in response to SARS-CoV-2 peptides in household contacts at T0 (n = 42) and T1 (n = 28) after whole-blood stimulation with 0.1 µg/mL of pools S **(A)** and M **(C)**, and 1 µg/mL of pool N **(B)**. Healthy donors (n=16) and COVID-19 patients (n = 53) were used as negative and positive control groups, respectively. The household contacts were stratified according to the swab result. The IFN-γ levels were assessed in plasma from stimulated whole-blood samples and reported by subtracting the background. The cut-off for each peptide pool was represented by a dashed line (pools S and N: 0.13 IU/mL; pool M: 0.19 IU/mL). Green triangle indicates the subject who scored positive only at T1. The red horizontal lines indicate the median. **(D, E)** Venn diagrams show the number of household contacts of COVID-19 cases at T0 and T1 with a positive response to the different SARS-CoV-2 peptides pools. **(F)** Venn diagrams show the number of confirmed hospitalized COVID-19 patients with a positive response to the different SARS-CoV-2 peptides pools, stratifying the results also with respect to days from symptom onset. The statistical comparison was done with the Kruskal-Wallis test and the Dunn’s multiple comparisons test, and p<0.05 was considered significant. IFN, interferon; COVID-19, COronaVIrus Disease 2019; S, spike; N, nucleocapsid; M, membrane.

The adaptive immune response includes both cell-mediated and humoral response. Regarding the cell-mediated response, the positivity rate was evaluated considering any positive T-cell response regardless of the peptides used. In the household contacts tested at T0, we found a specific T-cell response in 50% (5/10) of swab positive subjects. All these individuals responded to pool S, 3 of them scored positive also to N-specific stimulation, while only 1 individual tested positive to N-, M- and S-specific stimulations (**Figures 3A-D**). Among subjects with negative swab (n=32), 4 individuals (12.5%) showed a specific T-cell response. In particular, all subjects responded to pool S, whereas 2/4 to pools N or M (**Figure 3D** **, right panel**).

At T1, the specific T-cell response to SARS-CoV-2 peptide pools was detected in most of the swab positive subjects (77.8%, 7/9) (**Figures 3A-C** **)**, of whom all subjects responded to pool S, two individuals to both pools S and N and four subjects to all three peptide pools (**Figure 3E** **, left panel**). Among subjects with negative swab (n=19), five individuals (26.3%) showed a specific T-cell response (**Figure 3E** **, right panel**). In particular, three subjects responded to pool S, 1/5 to pool N and 4/5 to pool M.

In the “NO COVID-19” group the response to all three peptide pools was absent in most of the subjects (14/16, 87.5%), indicating a good accuracy of this test to discriminate “NO COVID-19” subjects from COVID-19 patients (pool S: p=0.0062; pool N: p=0.0043; pool M: p=0.034) (**Figures 3A-C**). In the COVID-19 cohort, the specific T-cell response to SARS-CoV-2 peptide pools was detected in 75.5% (40/53) of the individuals. Half of them responded to all three peptide pools regardless of the number of days elapsed since the onset of symptoms (**Figure 3F**). To note, the percentage of positive T-cell responders among COVID-19 patients was similar to the percentage observed in swab positive household contacts at T1 (7-20 days from the first swab) (**Figure 3E** **left panel**). Differently, at T0 most of the responders scored positive for both pools S and N, but not for pool M (**Figure 3D** **left panel**).

Regarding the quantitative response, significant differences were observed between IFN-γ levels of COVID-19 patients and those detected at T0 in the swab negative cohort of household contacts in response to all three peptide pools (p<0.0001 for both pools S and N, p=0.0058 for pool M). These differences persisted also at T1 for pools S and N (p=0.014 and p=0.013, respectively) (**Figures 3A-C**). On the contrary, the magnitude of the IFN-γ-specific response to all peptide pools was not significantly different between COVID-19 patients and swab positive household contacts (p>0.05).

### The serological response was detected in a minority of household contacts

**Figure 4:**
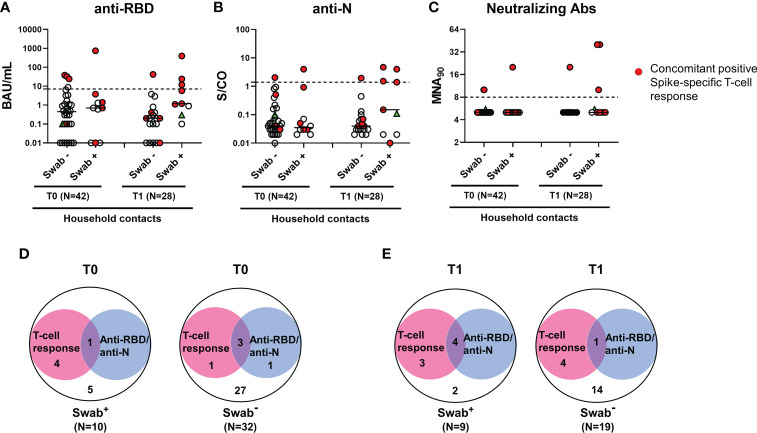
Antibody response in household contacts of COVID-19 cases. Evaluation of SARS-CoV-2-specific anti-RBD **(A)**, anti-N **(B)** and neutralizing **(C)** antibodies in household contacts at T0 (n=42) and T1 (n=28). Anti-RBD, anti-N and neutralizing antibodies were evaluated in sera samples and reported as Binding Antibody Units (BAU)/mL **(A)**, Sample/Cutoff (S/CO) **(B)**, and reciprocal of dilution (MNA_90_) **(C)**, respectively. Red dots indicate subjects with also a concomitant IFN-γ specific response as shown in the figure legend. Green triangle indicates the subject with positive swab only at T1. Dashed lines indicate the cut-off (anti-RBD: 7.1 BAU/mL; anti-N: 1.4 (S/CO); MNA_90_: 8). The black horizontal lines indicate the median. **(D, E)** Venn diagrams show the number of household contacts of COVID-19 cases at T0 and T1 with an IFN-γ response and/or the serological one (anti-RBD and anti-N). Statistical analysis was performed using Fisher’s exact test with Bonferroni correction and p<0.01 was considered significant. IFN, interferon; COVID-19, COronaVIrus Disease 2019; RBD, receptor binding domain; N, nucleocapsid.

Regarding the serological response, the positivity rate was evaluated considering any antibody response detected regardless of the antibody type considered. Only a minority of household contacts with positive swab showed anti-RBD/anti-N/neutralizing antibodies at baseline (1/10, 10%) (**Figures 4A-C**). Among individuals scored negative for the swab, 4 were positive for the serology (4/32, 12.5%): 3 subjects scored positive to both serological and cell-mediated responses, whereas one individual had only anti-RBD antibodies (**Figure 4D**). Among the total 5 subjects with detectable RBD/anti-N antibodies at T0, the neutralizing antibodies were detected in 3/5, of whom 2 were swab negative (**Figure 4C**). At T1, the serological response was still found only in a minority of swab positive subjects (44.4%, 4/9) (**Figure 4E**). No significant different proportions of antibody responders were found between swab positive subjects and negative ones (**Figure 4**).

Samples of the three seroconverted patients with positive swab were collected after 7 days, 14 days and 20 days from T0. For the other patients that did not seroconvert, samples were collected at 8 days (for 2 subjects), 10 days (for 2 subjects) and 18 days (for one individual) from T0. Due to the similarity of the time range, we cannot correlate the seroconversion score to the collection time. In the subjects analysed, the seroconversion score probably depends on the individual variability.

In particular, 3 subjects had both anti-RBD and anti-N antibodies, whereas one showed only anti-N antibodies. Neutralizing antibodies were detected in all 4 subjects with anti-RBD antibodies at T1, of whom one was also swab negative (**Figure 4C**).

To verify the SARS-CoV-2 specificity of the T-cell and serological responses observed in swab negative subjects, an indirect immunofluorescence assay was performed. The immunofluorescence IgG, IgM or IgA data revealed that subjects scored positive for the T-cell response but not for the serology (T0: n=1 and T1: n=4, see **Figures 4D, E** **)**, were confirmed negative for antibody response (**Supplementary Figure S5**). Therefore, the T-cell response detected in these swab negative subjects is probably due to a cross-reactivity with other cold coronaviruses. By contrast, in subjects scored positive for both T-cell response and serology (T0: n=3 and T1: n=1, see **Figures 4D, E** **)**, the immunofluorescence resulted positive. To note, plasmatic IFN-α was undetectable in these individuals. Therefore, the results suggest that these subjects may have had a previous SARS-CoV-2 infection.

### Kinetics of the immune responses in household contacts

To evaluate the kinetics of humoral- and cell-mediated immune responses to SARS-CoV-2 in the household contacts of COVID-19 cases, we compared the immune responses at T0 and T1 in 28 subjects longitudinally sampled. We observed that the number of responders to SARS-CoV-2 peptide pools, in particular to pools S and N, increased from T0 (4/8, 50%) to T1 (7/9, 77.8%) among swab positive subjects, although the difference was not significant (p=0.335) (**Table 4**). The same trend was also observed for the antibody response, although the total number of positive responders was still a minority (T0: 1/8, 12.5% vs T1: 4/9, 44.4%) compared to the T-cell response. A significant increase of the magnitude of the SARS-CoV-2 IFN-γ-N-specific response was observed at T1 compared to T0 (T0 median: 0.01 IU/mL, IQR: 0.01-0.02 vs T1 median: 0.035 IU/mL, IQR: 0.01-0.262, p=0.042) (**Supplementary Figure S6**). The same trend was also observed for the IFN-γ response to pool S (T0 median: 0.01 IU/mL, IQR: 0.01-0.047 vs T1 median: 0.035 IU/mL, IQR: 0.01-1.103, p=0.053), although not significant. Neither the quantitative IFN-γ-M-response nor the humoral one (anti-RBD or anti-N) showed significant differences comparing T0 and T1 (p=0.318, p=0.426 and p=0.407, respectively) (**Supplementary Figures S6C-E**).

**Table 4:** T-cell and antibody responses in household contacts of COVID-19 patients evaluated at both time points (T0 and T1).

		T0			T1			
		Positive swab N = 8	Negative swab N = 20	Tot N =28	Positive swab N = 9	Negative swab N = 19	Tot N = 28	P value*
**Positive T-cell responders** **N over total**		4 (50)	2 (10)	6 (21.4)	7 (77.8)	5 (26.3)	12 (42.8)	0.335
	**Pool S responders** [N (% among positive T-cell responders)]	4 (100)	2 (100)	6 (100)	7 (100)	3 (60)	10 (83.3)	0.335
** **	**Pool N responders** [N (% among positive T-cell responders)]	3 (75)	1 (50)	4 (66.7)	6(85.7)	1 (20)	7 (58.3)	0.345
** **	**Pool M responders** [N (% among positive T-cell responders)]	1 (25)	2 (100)	3(50)	4 (57.1)	4 (80)	8 (66.7)	0.294
**Positive antibody responders** **N over total**		1 (12.5)	1(5)	2 (7.1)	4 (44.4)	1 (10.5)	5 (41.7)	0.606
** **	**anti-RBD responders** [N (% among positive T-cell responders)]	1 (100)	1 (100)	2 (100)	3 (75)	1 (100)	4 (80)	0.576
	**anti-N responders** [N (% among positive T-cell responders)]	1 (100)	1 (100)	2 (100)	4 (100)	1 (100)	5 (100)	0.606

S, spike; N, nucleocapsid; M, membrane; RBD, receptor binding domain. *Chi-square test calculated among swab positive responders at the two time points.

Overall, the swab result showed a moderate concordance with the T-cell response (78.6%, k=0.467; with at T0: 78.6%, k=0.388 and at T1: 78.6%, k=0.536), and a scarce concordance with the serological response (72.9%, k=0.194 with at T0: 69%, k<1 and at T1: 78.6%, k=0.444).

## Discussion

A better understanding of the host immune responses to natural SARS-CoV-2 infection is critical to understand in depth the mechanisms of an effective host-defence in naïve unvaccinated populations. The results of these investigations can be useful to find new strategies for diagnosis and therapy. In this study, we investigated both the innate and adaptive immune responses in household contacts of COVID-19 cases followed over time to characterize the early immune responses to SARS-CoV-2 infection and their kinetics. By studying the innate immune response and the two compartments of adaptive immunity, T and B cells, we observed that each component of the SARS-CoV-2 immune response exhibited a distinct kinetic.

One primary function of the innate immune system during viral infection is limiting viral replication by inducing an inflammatory response. Type I IFNs, mainly consisting of IFN-α and IFN-β, represent the first rapid defensive line against invading pathogens, being important regulators of the adaptive immune response.

In the present study, we showed that the innate factor IFN-α is rapidly induced in all SARS-CoV-2-infected subjects at early stage of infection (T0). These patients were characterized by an asymptomatic or mild COVID-19. After 7-20 days (T1), IFN-α quickly disappeared. This data agrees with the type I IFN response detected by a blood transcriptome analysis in a cohort of subjects recently exposed to SARS-CoV-2 (42). We extended the analysis to a large panel of soluble factors identifying other cytokines/chemokines upregulated (IP-10, IL-1ra, MCP-1) or downregulated (IL-9, IL-1β, RANTES, MIP-1β) at the earliest stage of infection in household contacts with a positive swab. Among the cytokines/chemokines significantly modulated, IFN-α discriminated infected individuals from non-infected subjects with the highest accuracy.

Innate immunity mediated by type I IFN responses contributes to the protection against critical illness in COVID-19 (20–22). Indeed, SARS-CoV-2 has evolved several strategies to evade antiviral innate immune responses by reducing type I IFN levels acting at post-transcriptional level (49). In this regard, low serum levels of IFN-α have been reported in severe COVID-19 patients (50, 51) and associated with older age (52). Ineffective IFN-mediated innate immunity, due to neutralizing autoantibodies to type I IFNs and genetic polymorphisms causing a reduced expression of type I IFN receptor or inducible genes, has been strongly associated with inability to control the primary SARS-CoV-2 infection and a high risk of fatal COVID-19. In addition, the innate cell immunopathology and a plasma cytokine signature characterized by elevated IP-10, IL-6, and IL-8 levels have been also reported (18, 19, 53–57).

The up-regulated levels of IP-10, IL-1ra and MCP-1 observed in swab positive subjects are supported also by other findings showing that the levels of these cytokines/chemokines are prominent during the second week after disease onset and are even more pronounced in severe patients (19, 58, 59). The pro-inflammatory chemokine IP-10 and the monocyte chemoattractant factor MCP-1 contribute to the excessive inflammatory and immune response, favoring the recruitment of monocytes, macrophages, and T cells to the infection sites. The higher levels of IL-1ra found in the swab positive cohort are probably the consequence of the inflammatory process in progress. In this context, IL-1ra, as an early inhibitory immune factor, acts by controlling the inflammatory response. Serum concentrations of IL-1ra associated with COVID-19 severity. In particular, much higher levels of IL-1ra were observed in severe cases, indicating the presence of an overactive immune response (60, 61). The lower levels of the pro-inflammatory factors IL-1β, RANTES and MIP-1β, and of IL-9, a cytokine with direct and indirect effects on multiple cell types that affect the development of immunity and inflammation, may be indicative in our study cohort of a controlled inflammatory process, in which the IL-1ra exerts an effective action. Indeed, a higher production of IL-1β, RANTES, MIP-1β and IL-9 have been found in the severe disease (18, 59, 62). The differences, in terms of cytokine amount, observed between swab positive and negative subjects in our cohort are highly significant (at least p=0.007) for certain immune factors (IFN-α, IL-1ra, IP-10, IL-9 and MIP-1β). Moreover, the cytokine levels detected are comparable with what was reported in mild COVID-19 (61, 63). The cytokine profile observed might be indicative of the ongoing immune response to SARS-CoV-2 infection that distinguishes swab positive subjects from negative ones. Certainly, what we detect in the plasma is only a partial mirror of what happens in the respiratory tract, which is the target tissue of the virus. Further longitudinal studies on a larger cohort of subjects early exposed to COVID-19 and with different disease outcomes would be important to learn more about.

Regarding the adaptive immune response, it has been reported that CD4^+^ and CD8^+^ T-cell responses appear early after infection (32, 36) or vaccination (64–67), cross-recognize viral variants (10, 68), are over time stable and persist in vulnerable populations, albeit with a low amount (37, 38). In this manuscript, we showed that the T-cell response evaluated by a simple IGRA method based on the stimulation of whole blood with SARS-CoV-2 peptides from the S-, N-, or M-region, appears simultaneously or later compared to the innate immunity. It also increases over time becoming detectable in the majority of infected subjects (77.8%) after 7-20 days from the first swab. Moreover, the percentage of T-cell responders and the magnitude of the response in swab positive household contacts at T1 was similar to what was observed in the cohort of COVID-19 patients (75.5%).

Among the peptides tested, the best stimuli were those for the S- and N-region that detected the greater number of responders among the infected subjects. On the contrary, other works identified M- and N-related immunodominant peptides as the most effective in detecting the T-cell response. Discrepancies with our findings could be explained by the different methodologies (IGRA method vs flow cytometry analysis), populations analysed (household contacts early exposed vs COVID-19 convalescent and not convalescent patients), as well as the peptide format used (32, 69).

It is known that plasmatic type I IFN levels can be also detectable in response to acute respiratory infections different from SARS-CoV-2 (70). Differently, the IFN-γ response detected in the present study was specifically induced *in vitro* in response to SARS-CoV-2 peptides. However, the detection of a T-cell response also in some subjects with a negative swab and serology (5 five subjects in our household cohort) could be ascribed: i) to a cross-reactivity probably arising from previous seasonal coronavirus exposure, ii) or to previous exposure to SARS-CoV-2 without seroconversion or subsided antibody titers. The presence of SARS-CoV-2-specific T-cell response, whether due to SARS-CoV-2 infection or cross-reactivity, might explain the mild symptoms in infected household contacts and the resistance of other contacts to symptomatic SARS-CoV-2 infection (71).

By contrast, the humoral response was delayed by 1-2 weeks compared to the T-cell response, and it was detectable only in a minority (44%) of household contacts with confirmed infection. To note, the antibody response was further assessed for its ability to neutralize the virus. In this context, neutralizing antibodies were detectable in the few subjects scored positive for the serological response.

SARS-CoV-2 antibodies are detected later or can be absent in patients with less severe forms of COVID-19 (27–29) and their titers are not constant over time (31, 38, 72). Therefore, the IFN-γ T-cell specific response is important for viral containment (8) and potentially useful for the detection of infected subjects, even more in the context of the emerging variants that escape the antibody response (73, 74).

The present study is unique in terms of clinical cohort studied. In literature, the immune response in household contacts of COVID-19 cases was studied at only one time point (42, 71, 75, 76). Differently, our enrolled subjects were analysed at two time points (i.e., at the first nasopharyngeal swab and after 7-20 days), and with an easy-to-use assay to detect the T-cell specific response discriminating among the responses to N, M and S peptides. Moreover, both innate and adaptive immunity were evaluated and correlated to the SARS-CoV-2 viral load detected in swab specimens. In this respect, we showed that both plasma IFN-α and IP-10 levels were strongly associated with the viral load in swab specimens. Moreover, the swab test showed a moderate concordance with the T-cell response (78.6%, k=0.467), whereas a scarce concordance with the serological response (72.9%, k=0.194).

Some limitations of the study need to be considered. Firstly, the number of the enrolled household contacts was relatively small (42 subjects). This is due to the increasing uptake of the vaccination campaign that made more difficult the enrolment of unvaccinated individuals. However, the results are robust and in agreement with the recent findings generated in a larger cohort (42). Secondly, SARS-CoV-2 infections included in this study were likely caused by the Alpha variant, dominant between February and June 2021. Therefore, innate and adaptive immune responses may differ in timing and magnitude for the current and future variants.

Another important consideration needs to be made regarding the vaccination. In countries with high vaccination coverage, the T-cell specific response against pool S or anti-RBD antibodies cannot be longer used to discriminate infected and not-infected subjects, therefore the T-cell response to pool N might be a supporting approach for the diagnosis (9, 77).

In conclusion, we showed that household contacts with positive swab for SARS-CoV-2 present detectable plasmatic IFN-α and a viral–specific T-cell response, even in the absence of seroconversion, thus representing better indicators of SARS-Co-V-2 exposure than antibodies. The results of our exploratory study underline the role of plasmatic IFN-α and viral–specific T-cell response for a better understanding of the early immunological kinetic and for epidemiological studies.

## Data Availability Statement

The raw data supporting the conclusions of this article will be made available by the authors, without undue reservation.

## Ethics Statement

The Ethical Committee of the National Institute for Infectious Diseases (INMI) L. Spallanzani approved the study (approval number 247/2021). The patients/participants provided their written informed consent to participate in this study.

## Author Contributions

DG wrote the project to be submitted to the Ethical Committee. DG and EDR conceived and designed the study. Experiments for evaluating Innate and T cell response were performed by AA, VV, RC, AS, AMGA. Experiments for evaluating antibody response were performed by SM. AA performed the statistical analysis. AG, GC, LPi, MM, TAB, GG, and FP enrolled subjects and collected clinical data. AA and DG drafted the article and AG, MM, LPe, LPi, GG, CC, CA, EG, FP, EN, and EDR revised it critically. All authors critically analysed, discussed and interpreted data, and contributed to the article and approved the submitted version.

## Funding

This work was supported by funds 5x1000 and INMI “Lazzaro Spallanzani” COVID-2020-12371675 and Ricerca Corrente Linea 1 on emerging infections both funded by the Italian Ministry of Health and by generous liberal donations funding for COVID-19 research from Esselunga S.p.A., Camera di Commercio, Industria e Artigianato di Roma, Società Numero Blu Servizi S.p.A., Fineco Bank S.p.A., Associazione magistrati della Corte dei conti, and Società Mocerino Frutta Secca s.r.l. (resolutions n° 395 of May 25, 2021, n°254 of April 24, 2021, and n°257 of April 14, 2021). The funders were not involved in the study design, collection, analysis, and interpretation of data, the writing of this article, or the decision to submit it for publication.

## Acknowledgments

The authors gratefully acknowledge the nurse Veronica Caruso of the Local Public Health Office, ASL Roma 1, the study patients, and the collaborators of the National Institute for Infectious Diseases (INMI).

## Conflict of Interest

EG has received grants form Gilead and Mylan. EN is a member of the advisory board by Gilead, Lilly and Roche and received fees for educational training by Gilead, Lilly and Roche. DG is a member of the advisory board by Biomerieux and Eli-Lilly, and received fees for educational training or consultancy by Biogen, Cellgene, Diasorin, Janssen, Qiagen, Quidel.

The remaining authors declare that the research was conducted in the absence of any commercial or financial relationships that could be construed as a potential conflict of interest.

## Publisher’s Note

All claims expressed in this article are solely those of the authors and do not necessarily represent those of their affiliated organizations, or those of the publisher, the editors and the reviewers. Any product that may be evaluated in this article, or claim that may be made by its manufacturer, is not guaranteed or endorsed by the publisher.

## References

[1] Wu F Liu M Wang A Lu L Wang Q Gu C. Evaluating the association of clinical characteristics with neutralizing antibody levels in patients who have recovered from mild COVID-19 in shanghai, China. JAMA Intern Med (2020) 180:1356–62. doi: 10.1001/jamainternmed.2020.4616 PMC9377417 32808970

[2] Guan W-J Ni Z-Y Hu Y Liang W-H Ou C-Q He J-X. Clinical characteristics of coronavirus disease 2019 in China. N Engl J Med (2020) 382:1708–20. doi: 10.1056/NEJMoa2002032 PMC7092819 32109013

[3] Wölfel R Corman VM Guggemos W Seilmaier M Zange S Müller MA. Virological assessment of hospitalized patients with COVID-2019. Nature (2020) 581:465–9. doi: 10.1038/s41586-020-2196-x 32235945

[4] Wu Z McGoogan JM. Characteristics of and important lessons from the coronavirus disease 2019 (COVID-19) outbreak in China: Summary of a report of 72 314 cases from the Chinese center for disease control and prevention. JAMA (2020) 323:1239–42. doi: 10.1001/jama.2020.2648 32091533

[5] Merad M Martin JC. Pathological inflammation in patients with COVID-19: a key role for monocytes and macrophages. Nat Rev Immunol (2020) 20:355–62. doi: 10.1038/s41577-020-0331-4 PMC7201395 32376901

[6] Vabret N Britton GJ Gruber C Hegde S Kim J Kuksin M. Immunology of COVID-19: Current state of the science. Immunity (2020) 52:910–41. doi: 10.1016/j.immuni.2020.05.002 PMC7200337 32505227

[7] Marshall M. The lasting misery of coronavirus long-haulers. Nature (2020) 585:339–41. doi: 10.1038/d41586-020-02598-6 32929257

[8] Sette A Crotty S. Adaptive immunity to SARS-CoV-2 and COVID-19. Cell (2021) 184:861–80. doi: 10.1016/j.cell.2021.01.007 PMC7803150 33497610

[9] Goletti D Petrone L Manissero D Bertoletti A Rao S Ndunda N. The potential clinical utility of measuring severe acute respiratory syndrome coronavirus 2-specific T-cell responses. Clin Microbiol Infect Off Publ Eur Soc Clin Microbiol Infect Dis (2021) 27:1784–9. doi: 10.1016/j.cmi.2021.07.005 PMC8272618 34256141

[10] Tarke A Coelho CH Zhang Z Dan JM Yu ED Methot N. SARS-CoV-2 vaccination induces immunological T cell memory able to cross-recognize variants from alpha to omicron. Cell (2022) 185:847–859.e11. doi: 10.1016/j.cell.2022.01.015 35139340 PMC8784649

[11] Ferraccioli G Gremese E Goletti D Petrone L Cantini F Ugel S. Immune-guided therapy of COVID-19. Cancer Immunol Res (2022) 10:384–402. doi: 10.1158/2326-6066.CIR-21-0675 35074758

[12] Schultze JL Aschenbrenner AC. COVID-19 and the human innate immune system. Cell (2021) 184:1671–92. doi: 10.1016/j.cell.2021.02.029 PMC7885626 33743212

[13] Petrone L Petruccioli E Vanini V Cuzzi G Najafi Fard S Alonzi T. A whole blood test to measure SARS-CoV-2-specific response in COVID-19 patients. Clin Microbiol Infect Off Publ Eur Soc Clin Microbiol Infect Dis (2021) 27:286.e7–286.e13. doi: 10.1016/j.cmi.2020.09.051 PMC7547312 33045370

[14] Petrone L Petruccioli E Alonzi T Vanini V Cuzzi G Najafi Fard S. In-vitro evaluation of the immunomodulatory effects of baricitinib: Implication for COVID-19 therapy. J Infect (2021) 82:58–66. doi: 10.1016/j.jinf.2021.02.023 33639176 PMC7904476

[15] Weiskopf D Schmitz KS Raadsen MP Grifoni A Okba NMA Endeman H. Phenotype and kinetics of SARS-CoV-2-specific T cells in COVID-19 patients with acute respiratory distress syndrome. Sci Immunol (2020) 5:eabd2071. doi: 10.1126/sciimmunol.abd2071 32591408 PMC7319493

[16] De Biasi S Meschiari M Gibellini L Bellinazzi C Borella R Fidanza L. Marked T cell activation, senescence, exhaustion and skewing towards TH17 in patients with COVID-19 pneumonia. Nat Commun (2020) 11:3434. doi: 10.1038/s41467-020-17292-4 32632085 PMC7338513

[17] Blanco-Melo D Nilsson-Payant BE Liu W-C Uhl S Hoagland D Møller R. Imbalanced host response to SARS-CoV-2 drives development of COVID-19. Cell (2020) 181:1036–1045.e9. doi: 10.1016/j.cell.2020.04.026 32416070 PMC7227586

[18] Del Valle DM Kim-Schulze S Huang H-H Beckmann ND Nirenberg S Wang B. An inflammatory cytokine signature predicts COVID-19 severity and survival. Nat Med (2020) 26:1636–43. doi: 10.1038/s41591-020-1051-9 PMC7869028 32839624

[19] Lucas C Wong P Klein J Castro TBR Silva J Sundaram M. Longitudinal analyses reveal immunological misfiring in severe COVID-19. Nature (2020) 584:463–9. doi: 10.1038/s41586-020-2588-y PMC7477538 32717743

[20] Bastard P Rosen LB Zhang Q Michailidis E Hoffmann H-H Zhang Y. Autoantibodies against type I IFNs in patients with life-threatening COVID-19. Science (2020) 370:eabd4585. doi: 10.1126/science.abd4585 32972996 PMC7857397

[21] Pairo-Castineira E Clohisey S Klaric L Bretherick AD Rawlik K Pasko D. Genetic mechanisms of critical illness in COVID-19. Nature (2021) 591:92–8. doi: 10.1038/s41586-020-03065-y 33307546

[22] Zhang Q Bastard P Liu Z Le Pen J Moncada-Velez M Chen J. Inborn errors of type I IFN immunity in patients with life-threatening COVID-19. Science (2020) 370:eabd4570. doi: 10.1126/science.abd4570 32972995 PMC7857407

[23] Hotez PJ Corry DB Strych U Bottazzi ME. COVID-19 vaccines: neutralizing antibodies and the alum advantage. Nat Rev Immunol (2020) 20:399–400. doi: 10.1038/s41577-020-0358-6 32499636 PMC7271131

[24] Robbiani DF Gaebler C Muecksch F Lorenzi JCC Wang Z Cho A. Convergent antibody responses to SARS-CoV-2 in convalescent individuals. Nature (2020) 584:437–42. doi: 10.1038/s41586-020-2456-9 PMC7442695 32555388

[25] Seydoux E Homad LJ MacCamy AJ Parks KR Hurlburt NK Jennewein MF. Analysis of a SARS-CoV-2-Infected Individual Reveals Development of Potent Neutralizing Antibodies with Limited Somatic Mutation. Immunity (2020) 53:98–105.e5. doi: 10.1016/j.immuni.2020.06.001 PMC7276322 32561270

[26] Wang C Li W Drabek D Okba NMA van Haperen R Osterhaus ADME. A human monoclonal antibody blocking SARS-CoV-2 infection. Nat Commun (2020) 11:2251. doi: 10.1038/s41467-020-16256-y 32366817 PMC7198537

[27] Long Q-X Liu B-Z Deng H-J Wu G-C Deng K Chen Y-K. Antibody responses to SARS-CoV-2 in patients with COVID-19. Nat Med (2020) 26:845–8. doi: 10.1038/s41591-020-0897-1 32350462

[28] Mallapaty S. Will antibody tests for the coronavirus really change everything? Nature (2020) 580:571–2. doi: 10.1038/d41586-020-01115-z 32313159

[29] Woloshin S Patel N Kesselheim AS. False negative tests for SARS-CoV-2 infection - challenges and implications. N Engl J Med (2020) 383:e38. doi: 10.1056/NEJMp2015897 32502334

[30] Seow J Graham C Merrick B Acors S Pickering S Steel KJA. Longitudinal observation and decline of neutralizing antibody responses in the three months following SARS-CoV-2 infection in humans. Nat Microbiol (2020) 5:1598–607. doi: 10.1038/s41564-020-00813-8 PMC7610833 33106674

[31] Yamayoshi S Yasuhara A Ito M Akasaka O Nakamura M Nakachi I. Antibody titers against SARS-CoV-2 decline, but do not disappear for several months. EClinicalMedicine (2021) 32:100734. doi: 10.1016/j.eclinm.2021.100734 33589882 PMC7877219

[32] Grifoni A Weiskopf D Ramirez SI Mateus J Dan JM Moderbacher CR. Targets of T cell responses to SARS-CoV-2 coronavirus in humans with COVID-19 disease and unexposed individuals. Cell (2020) 181:1489–1501.e15. doi: 10.1016/j.cell.2020.05.015 32473127 PMC7237901

[33] Ni L Ye F Cheng M-L Feng Y Deng Y-Q Zhao H. Detection of SARS-CoV-2-Specific humoral and cellular immunity in COVID-19 convalescent individuals. Immunity (2020) 52:971–977.e3. doi: 10.1016/j.immuni.2020.04.023 32413330 PMC7196424

[34] Sekine T Perez-Potti A Rivera-Ballesteros O Strålin K Gorin J-B Olsson A. Robust T cell immunity in convalescent individuals with asymptomatic or mild COVID-19. Cell (2020) 183:158–168.e14. doi: 10.1016/j.cell.2020.08.017 32979941 PMC7427556

[35] Le Bert N Tan AT Kunasegaran K Tham CYL Hafezi M Chia A. SARS-CoV-2-specific T cell immunity in cases of COVID-19 and SARS, and uninfected controls. Nature (2020) 584:457–62. doi: 10.1038/s41586-020-2550-z 32668444

[36] Aiello A Najafi Fard S Petruccioli E Petrone L Vanini V Farroni C. Spike is the most recognized antigen in the whole-blood platform in both acute and convalescent COVID-19 patients. Int J Infect Dis IJID Off Publ Int Soc Infect Dis (2021) 106:338–47. doi: 10.1016/j.ijid.2021.04.034 PMC8045417 33864921

[37] Tortorella C Aiello A Gasperini C Agrati C Castilletti C Ruggieri S. Humoral- and T-Cell-Specific Immune Responses to SARS-CoV-2 mRNA Vaccination in Patients With MS Using Different Disease-Modifying Therapies. Neurology (2022) 98:e541–54. doi: 10.1212/WNL.0000000000013108 PMC8826460 34810244

[38] Farroni C Picchianti-Diamanti A Aiello A Nicastri E Laganà B Agrati C. Kinetics of the b- and T-cell immune responses after 6 months from SARS-CoV-2 mRNA vaccination in patients with rheumatoid arthritis. Front Immunol (2022) 13:846753. doi: 10.3389/fimmu.2022.846753 35309297 PMC8924958

[39] Petruccioli E Najafi Fard S Navarra A Petrone L Vanini V Cuzzi G. Exploratory analysis to identify the best antigen and the best immune biomarkers to study SARS-CoV-2 infection. J Transl Med (2021) 19:272. doi: 10.1186/s12967-021-02938-8 34174875 PMC8235902

[40] Picchianti-Diamanti A Aiello A Laganà B Agrati C Castilletti C Meschi S. ImmunosuppressiveTherapies differently modulate humoral- and T-Cell-Specific responses to COVID-19 mRNA vaccine in rheumatoid arthritis patients. Front Immunol (2021) 12:740249. doi: 10.3389/fimmu.2021.740249 34594343 PMC8477040

[41] Rydyznski Moderbacher C Ramirez SI Dan JM Grifoni A Hastie KM Weiskopf D. Antigen-specific adaptive immunity to SARS-CoV-2 in acute COVID-19 and associations with age and disease severity. Cell (2020) 183:996–1012.e19. doi: 10.1016/j.cell.2020.09.038 33010815 PMC7494270

[42] Chandran A Rosenheim J Nageswaran G Swadling L Pollara G Gupta RK. Rapid synchronous type 1 IFN and virus-specific T cell responses characterize first wave non-severe SARS-CoV-2 infections. Cell Rep Med (2022) 3:100557. doi: 10.1016/j.xcrm.2022.100557 35474751 PMC8895494

[43] Nicastri E Petrosillo N Ascoli Bartoli T Lepore L Mondi A Palmieri F. National institute for the infectious diseases “L. spallanzani”, IRCCS. recommendations for COVID-19 clinical management. Infect Dis Rep (2020) 12:8543. doi: 10.4081/idr.2020.8543 32218915 PMC7097833

[44] WHO. Clinical management of COVID-19: living guidance. World Health Organization (2021). 35917394

[45] Petrone L Petruccioli E Vanini V Cuzzi G Gualano G Vittozzi P. Coinfection of tuberculosis and COVID-19 limits the ability to *in vitro* respond to SARS-CoV-2. Int J Infect Dis IJID Off Publ Int Soc Infect Dis (2021) 113 Suppl 1:S82–7. doi: 10.1016/j.ijid.2021.02.090 PMC7944764 33713816

[46] Corman VM Landt O Kaiser M Molenkamp R Meijer A Chu DK. Detection of 2019 novel coronavirus (2019-nCoV) by real-time RT-PCR. Euro Surveill Bull Eur Sur Mal Transm Eur Commun Dis Bull (2020) 25:2000045. doi: 10.2807/1560-7917.ES.2020.25.3.2000045 PMC6988269 31992387

[47] Matusali G Colavita F Lapa D Meschi S Bordi L Piselli P. SARS-CoV-2 serum neutralization assay: A traditional tool for a brand-new virus. Viruses (2021) 13:655. doi: 10.3390/v13040655 33920222 PMC8069482

[48] Colavita F Biava M Castilletti C Lanini S Miccio R Portella G. Inflammatory and humoral immune response during Ebola virus infection in survivor and fatal cases occurred in Sierra Leone during the 2014^–^2016 outbreak in West Africa. Viruses (2019) 11:E373. doi: 10.3390/v11040373 31018522 PMC6520887

[49] Burke JM St Clair LA Perera R Parker R. SARS-CoV-2 infection triggers widespread host mRNA decay leading to an mRNA export block. RNA N Y N (2021) 27:1318–29. doi: 10.1261/rna.078923.121 PMC8522697 34315815

[50] Hadjadj J Yatim N Barnabei L Corneau A Boussier J Smith N. Impaired type I interferon activity and inflammatory responses in severe COVID-19 patients. Science (2020) 369:718–24. doi: 10.1126/science.abc6027 PMC7402632 32661059

[51] Contoli M Papi A Tomassetti L Rizzo P Vieceli Dalla Sega F Fortini F. Blood interferon-α levels and severity, outcomes, and inflammatory profiles in hospitalized COVID-19 patients. Front Immunol (2021) 12:648004. doi: 10.3389/fimmu.2021.648004 33767713 PMC7985458

[52] Alidjinou EK Hirabidian M Rabat A Ouafi M Nekoua MP Sane F. The lille covid research network licorne null. low serum levels of interferon alpha in COVID-19 patients are associated with older age. J Clin Med (2022) 11:961. doi: 10.3390/jcm11040961 35207234 PMC8877658

[53] Aid M Busman-Sahay K Vidal SJ Maliga Z Bondoc S Starke C. Vascular disease and thrombosis in SARS-CoV-2-Infected rhesus macaques. Cell (2020) 183:1354–1366.e13. doi: 10.1016/j.cell.2020.10.005 33065030 PMC7546181

[54] Kuri-Cervantes L Pampena MB Meng W Rosenfeld AM Ittner CAG Weisman AR. Comprehensive mapping of immune perturbations associated with severe COVID-19. Sci Immunol (2020) 5:eabd7114. doi: 10.1126/sciimmunol.abd7114 32669287 PMC7402634

[55] Li X Liu C Mao Z Xiao M Wang L Qi S. Predictive values of neutrophil-to-lymphocyte ratio on disease severity and mortality in COVID-19 patients: a systematic review and meta-analysis. Crit Care Lond Engl (2020) 24:647. doi: 10.1186/s13054-020-03374-8 PMC7667659 33198786

[56] Radermecker C Detrembleur N Guiot J Cavalier E Henket M d’Emal C. Neutrophil extracellular traps infiltrate the lung airway, interstitial, and vascular compartments in severe COVID-19. J Exp Med (2020) 217:e20201012. doi: 10.1084/jem.20201012 32926097 PMC7488867

[57] Schurink B Roos E Radonic T Barbe E Bouman CSC de Boer HH. Viral presence and immunopathology in patients with lethal COVID-19: a prospective autopsy cohort study. Lancet Microbe (2020) 1:e290–9. doi: 10.1016/S2666-5247(20)30144-0 PMC7518879 33015653

[58] Zhang Z Ai G Chen L Liu S Gong C Zhu X. Associations of immunological features with COVID-19 severity: a systematic review and meta-analysis. BMC Infect Dis (2021) 21:738. doi: 10.1186/s12879-021-06457-1 34344353 PMC8329624

[59] Hsu R-J Yu W-C Peng G-R Ye C-H Hu S Chong PCT. The role of cytokines and chemokines in severe acute respiratory syndrome coronavirus 2 infections(2022) (Accessed May 28, 2022). 10.3389/fimmu.2022.832394 PMC9021400 35464491

[60] Zhao Y Qin L Zhang P Li K Liang L Sun J. Longitudinal COVID-19 profiling associates IL-1RA and IL-10 with disease severity and RANTES with mild disease. JCI Insight (2020) 5:139834. doi: 10.1172/jci.insight.139834 32501293 PMC7406242

[61] Ling L Chen Z Lui G Wong CK Wong WT Ng RWY. Longitudinal cytokine profile in patients with mild to critical COVID-19. Front Immunol (2021) 12:763292. doi: 10.3389/fimmu.2021.763292 34938289 PMC8685399

[62] Grishaeva A Ponezheva Z Chanyshev M Ploskireva A Usenko D Tsvetkova N. MIP-1a and MIP-1b in serum as potential markers of the severe course COVID-19. Int J Infect Dis (2022) 116:S44. doi: 10.1016/j.ijid.2021.12.105

[63] Liu Y Zhang C Huang F Yang Y Wang F Yuan J. Elevated plasma levels of selective cytokines in COVID-19 patients reflect viral load and lung injury. Natl Sci Rev (2020) 7:1003–11. doi: 10.1093/nsr/nwaa037 PMC7107806 34676126

[64] Angyal A Longet S Moore SC Payne RP Harding A Tipton T. T-Cell and antibody responses to first BNT162b2 vaccine dose in previously infected and SARS-CoV-2-naive UK health-care workers: a multicentre prospective cohort study. Lancet Microbe (2022) 3:e21–31. doi: 10.1016/S2666-5247(21)00275-5 PMC8577846 34778853

[65] Agrati C Castilletti C Goletti D Meschi S Sacchi A Matusali G. Coordinate induction of humoral and spike specific T-cell response in a cohort of Italian health care workers receiving BNT162b2 mRNA vaccine. Microorganisms (2021) 9:1315. doi: 10.3390/microorganisms9061315 34208751 PMC8235087

[66] Petrone L Picchianti-Diamanti A Sebastiani GD Aiello A Laganà B Cuzzi G. Humoral and cellular responses to spike of δ SARS-CoV-2 variant in vaccinated patients with immune-mediated inflammatory diseases. Int J Infect Dis IJID Off Publ Int Soc Infect Dis (2022) 121:24–30. doi: 10.1016/j.ijid.2022.04.027 PMC9023365 35462039

[67] Petrone L Tortorella C Aiello A Farroni C Ruggieri S Castilletti C. Humoral and cellular response to spike of delta SARS-CoV-2 variant in vaccinated patients with multiple sclerosis(2022) (Accessed May 31, 2022). 10.3389/fneur.2022.881988 PMC9194677 35711277

[68] Gao Y Cai C Grifoni A Müller TR Niessl J Olofsson A. Ancestral SARS-CoV-2-specific T cells cross-recognize the omicron variant. Nat Med (2022) 28:472–6. doi: 10.1038/s41591-022-01700-x PMC8938268 35042228

[69] Thieme CJ Anft M Paniskaki K Blazquez-Navarro A Doevelaar A Seibert FS. Robust T cell response toward spike, membrane, and nucleocapsid SARS-CoV-2 proteins is not associated with recovery in critical COVID-19 patients. Cell Rep Med (2020) 1:100092. doi: 10.1016/j.xcrm.2020.100092 32904468 PMC7456276

[70] Liu T-Y Burke T Park LP Woods CW Zaas AK Ginsburg GS. An individualized predictor of health and disease using paired reference and target samples. BMC Bioinf (2016) 17:47. doi: 10.1186/s12859-016-0889-9 PMC4722633 26801061

[71] Gallais F Velay A Nazon C Wendling M-J Partisani M Sibilia J. Intrafamilial exposure to SARS-CoV-2 associated with cellular immune response without seroconversion, France. Emerg Infect Dis (2021) 27:113–21. doi: 10.3201/eid2701.203611 PMC7774579 33261718

[72] Sethuraman N Jeremiah SS Ryo A. Interpreting diagnostic tests for SARS-CoV-2. JAMA (2020) 323:2249–51. doi: 10.1001/jama.2020.8259 32374370

[73] Cameroni E Saliba C Bowen JE Rosen LE Culap K Pinto D. Broadly neutralizing antibodies overcome SARS-CoV-2 Omicron antigenic shift. Nature (2022) 602:664–70. doi: 10.1038/s41586-021-04386-2 PMC9531318 35016195

[74] Liu L Iketani S Guo Y Chan JF-W Wang M Liu L. Striking antibody evasion manifested by the omicron variant of SARS-CoV-2. Nature (2022) 602:676–81. doi: 10.1038/s41586-021-04388-0 35016198

[75] Murugesan K Jagannathan P Altamirano J Maldonado YA Bonilla HF Jacobson KB. Long term accuracy of SARS-CoV-2 interferon-γ release assay and its application in household investigation. Clin Infect Dis Off Publ Infect Dis Soc Am (2022), ciac045. doi: 10.1093/cid/ciac045 PMC8807306 35079772

[76] Neeland MR Bannister S Clifford V Nguyen J Dohle K Overmars I. Children and adults in a household cohort study have robust longitudinal immune responses following SARS-CoV-2 infection or exposure. Front Immunol (2021) 12:741639. doi: 10.3389/fimmu.2021.741639 34721408 PMC8548628

[77] Ogbe A Kronsteiner B Skelly DT Pace M Brown A Adland E. T Cell assays differentiate clinical and subclinical SARS-CoV-2 infections from cross-reactive antiviral responses. Nat Commun (2021) 12:2055. doi: 10.1038/s41467-021-21856-3 33824342 PMC8024333

