# Supplementary material for: Coordinated innate and T-cell immune responses in mild COVID-19 patients from household contacts of COVID-19 cases during the first pandemic wave
Source: Front Immunol. 2022 Jul 27;13:920227. doi: 10.3389/fimmu.2022.920227 (PMC9364317; doi:10.3389/fimmu.2022.920227)
Supplement: Supplementary file 1 [file DataSheet_1.docx]

Supplementary Material

| **Supplementary Table 1. Differences in the soluble factors detected in the plasma of household contacts at T0 vs T1.** | | |
| --- | --- | --- |
| **Cytokines/Chemokines** | **Swab negative** | **Swab positive** |
| IFN-α | 0.125 | 0.156 |
| IFN-β | 0.945 | >0.999 |
| IL-1β | 0.627 | 0.250 |
| IL-1ra | 0.137 | 0.375 |
| IL-2 | 0.105 | >0.999 |
| IL-4 | 0.926 | 0.437 |
| IL-5 | - | - |
| IL-6 | >0.999 | 0.625 |
| IL-7 | >0.999 | >0.999 |
| IL-8 | 0.246 | 0.062 |
| IL-9 | 0.719 | 0.093 |
| IL-10 | 0.582 | 0.125 |
| IL-12(p70) | 0.859 | - |
| IL-13 | >0.999 | >0.999 |
| IL-15 | - | - |
| IL-17A | 0.194 | 0.625 |
| Eotaxin | 0.561 | 0.312 |
| FGF basic | 0.375 | 0.500 |
| G-CSF | 0.061 | 0.062 |
| GM-CSF | 0.312 | >0.999 |
| IFN-γ | 0.197 | 0.562 |
| IP-10 | 0.679 | 0.312 |
| MCP-1 | 0.637 | 0.094 |
| MIP-1α | **0.008** | 0.062 |
| PDGF-bb | 0.599 | 0.437 |
| MIP-1β | 0.277 | 0.062 |
| RANTES | 0.421 | 0.437 |
| TNF-α | 0.323 | 0.312 |
| VEGF | 0.812 | >0.999 |
| Footnotes: the statistical analysis was performed using the Wilcoxon signed rank test. A p<0.05 was considered significant. In bold is reported the only significant value. | | |

**Supplementary Figure S1**

**
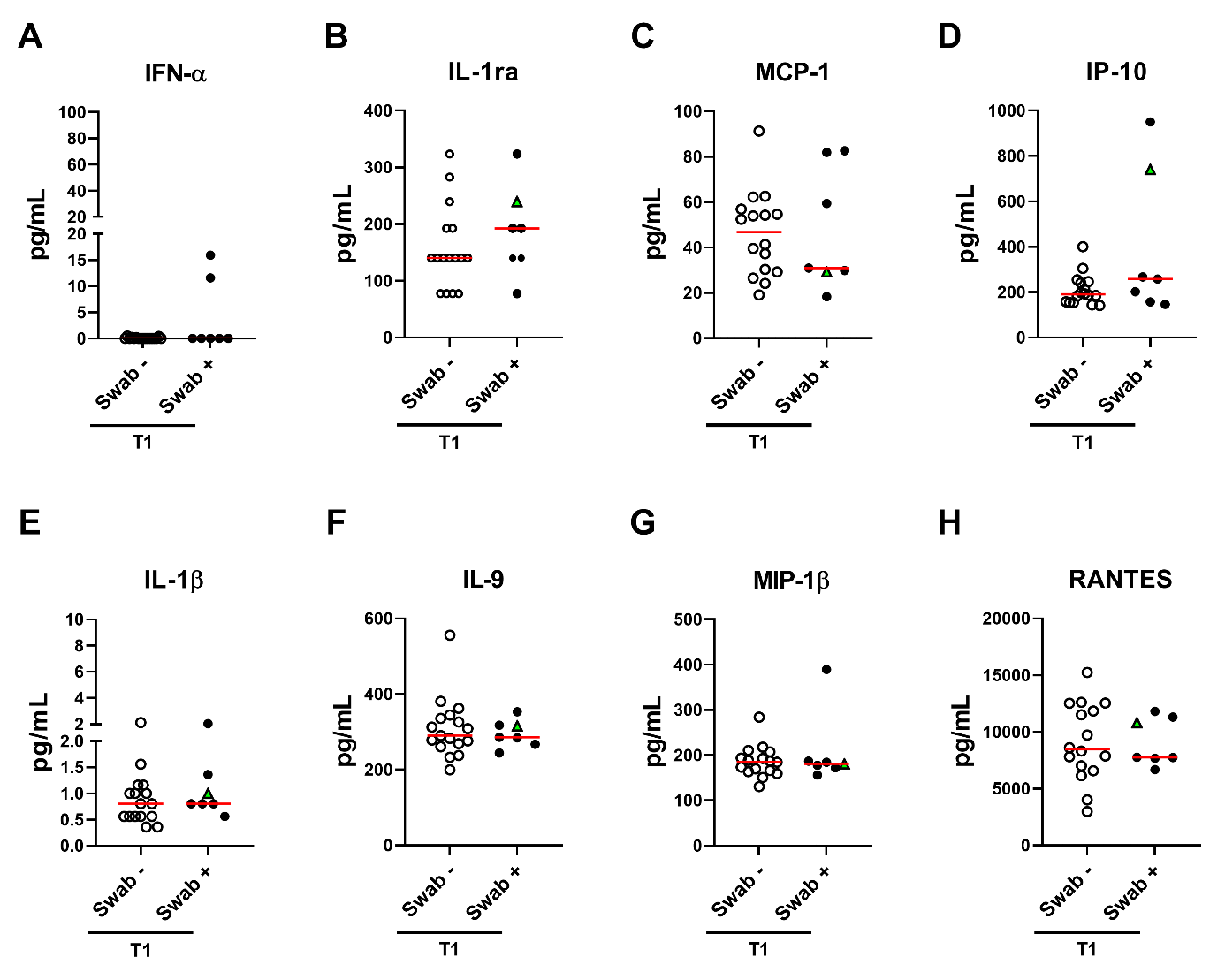
**

**Supplementary Figure 1. Plasmatic cytokines/chemokines modulated in household contacts at baseline but not at the follow-up.** (**A-H**) Household contacts at T1 (n=23) were stratified according to the swab result: positive (n=7) and negative (n=16). Plasma harvested from unstimulated blood samples were tested for the detection of 27 cytokines/chemokines using the Bio-Plex Pro Human Cytokine 27-plex Assay and for the detection of IFN-α and-β by means of an automatic ELISA. Red horizontal lines indicate medians. The green triangle identifies the subject with a positive swab only at T1. Statistical analysis was performed using Mann-Whitney U test to compare swab positive and negative subjects. A p<0.05 was considered significant. Footnotes: IL, interleukin; MCP, monocyte chemoattractant protein; MIP, macrophage inflammatory protein; IP, Interferon-gamma induced protein; RANTES, regulated on activation IFN, interferon.

**Supplementary Figure S2**

**
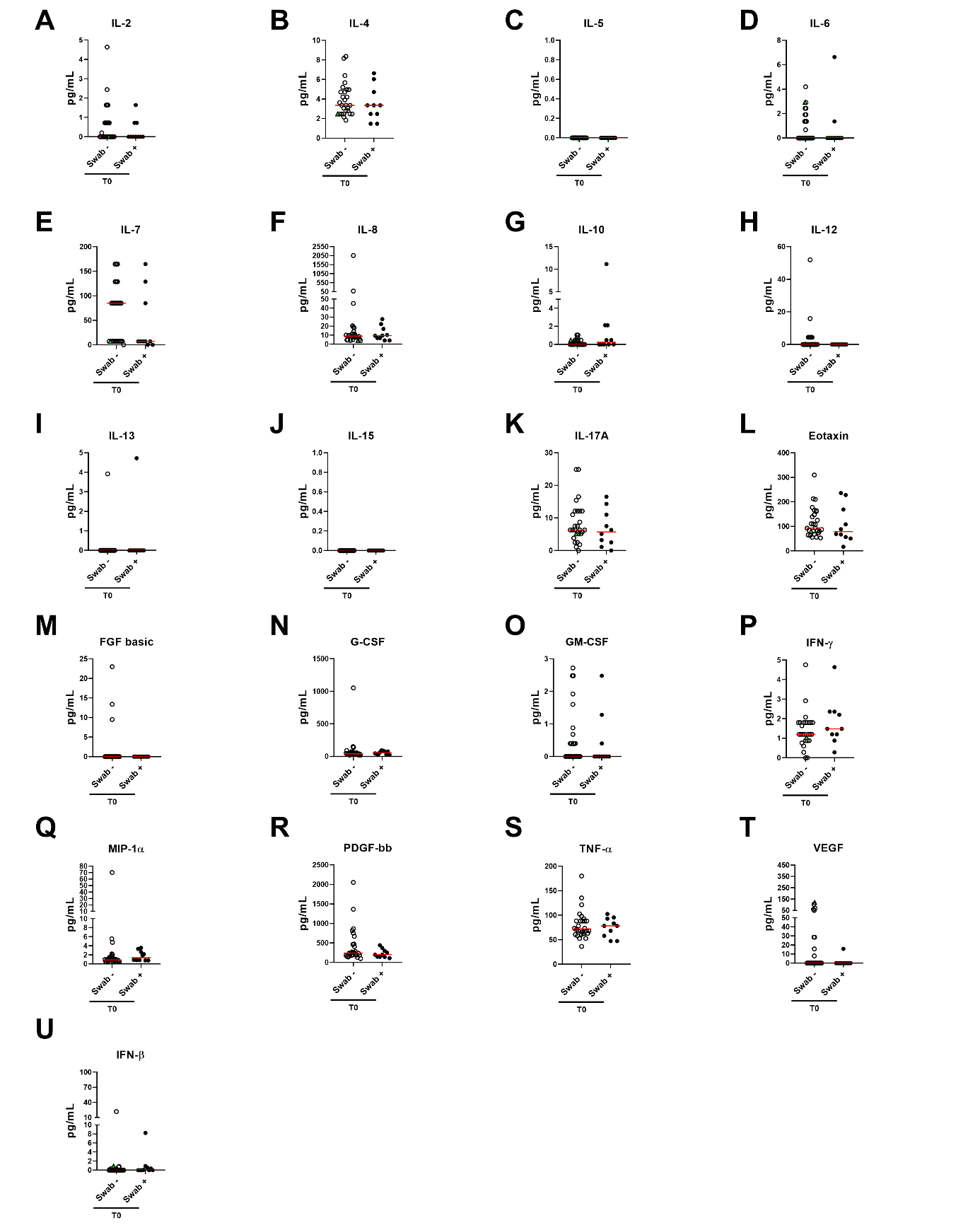
**

**Supplementary Figure 2. Plasmatic cytokines/chemokines not modulated in household contacts of COVID-19 subjects at T0. (A-U)** Household contacts at T0 (n=38) were stratified according to the swab result: positive (n=10) and negative (n=28). Red horizontal lines indicate medians. The green triangle identifies the subject with a positive swab only at T1. Statistical analysis was performed using Mann-Whitney U test to compare swab positive and negative subjects. p<0.05 was considered significant. Footnotes: IL, interleukin; FGF, basic fibroblast growth factor; G-CSF, granulocyte-colony stimulating factor; GM-CSF, granulocyte-macrophage colony-stimulating factor; MIP, macrophage inflammatory protein; PDGF, platelet-derived growth factor; TNF tumour necrosis factor; VEGF, vascular endothelial growth factor; IFN, interferon.

**Supplementary Figure S3**

**
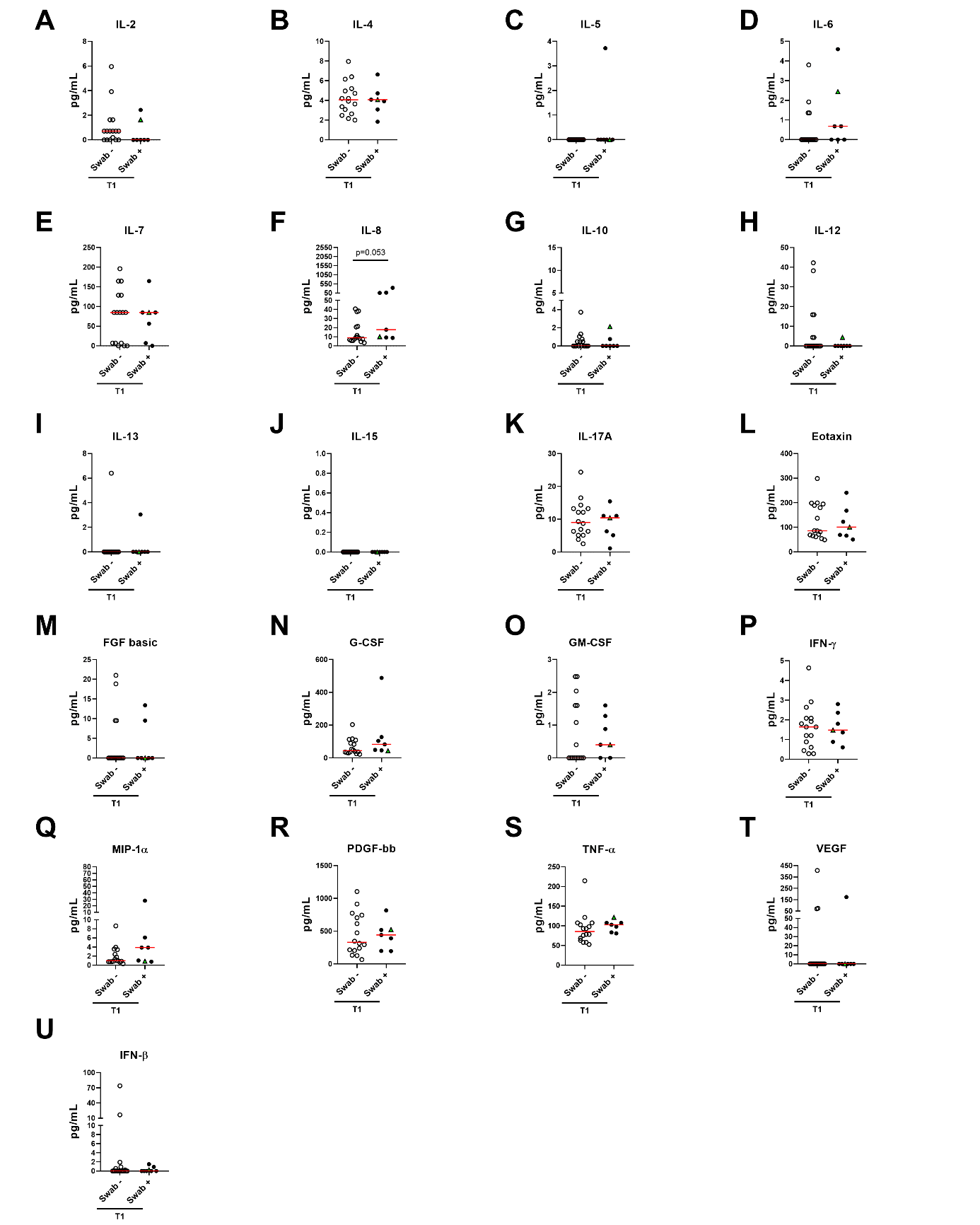
**

**Supplementary Figure 3. Plasmatic cytokines/chemokines analyzed in household contacts of COVID-19 subjects at T1. (A-U)** Household contacts at T1 (n=23) were stratified according to the swab result: positive (n=7) and negative (n=16). Red horizontal lines indicate medians. The green triangle identifies the subject with a positive swab only at T1. Statistical analysis was performed using Mann-Whitney U test to compare swab positive and negative subjects p<0.05 was considered significant. Footnotes: IL, interleukin; FGF, basic fibroblast growth factor; G-CSF, granulocyte-colony stimulating factor; GM-CSF, granulocyte-macrophage colony-stimulating factor; MIP, macrophage inflammatory protein; PDGF, platelet-derived growth factor; TNF tumour necrosis factor; VEGF, vascular endothelial growth factor; IFN, interferon.

**Supplementary Figure S4**

**
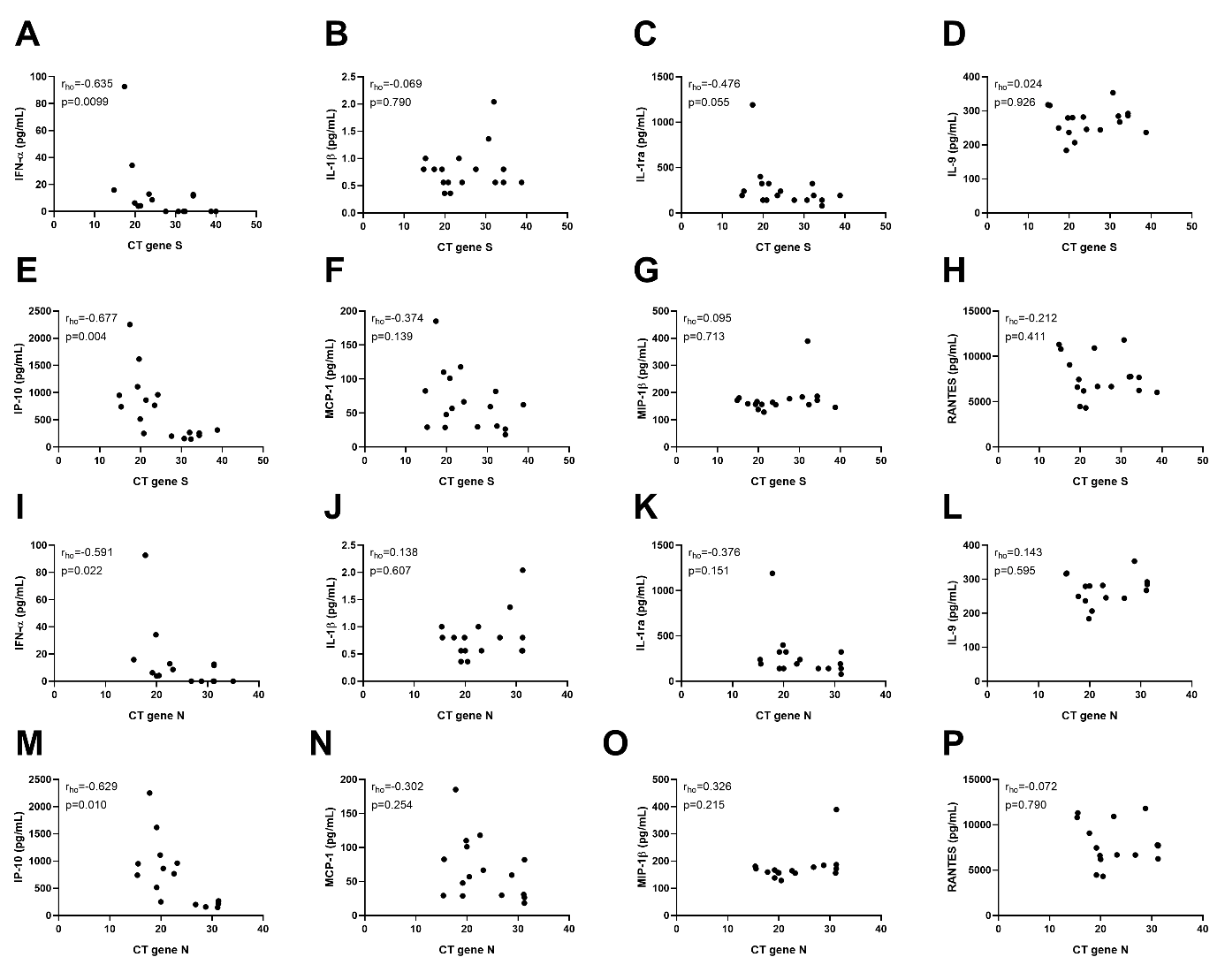
**

**Supplementary Figure 4. Significant negative correlations were found between the RT-PCR Cycle threshold (Ct) values reported for genes S and N and IFN-α or IP-10 levels.** Correlations between plasmatic cytokines/chemokines and Ct values of genes S (**A**-**H**) and N (**I**-**P**) within the cohort of swab positive subjects are reported. Cytokines/chemokines levels were measured in plasma harvested from unstimulated blood samples and expressed in pg/mL. Non-parametric Spearman's rank test was used to assess correlations (p<0.05). Footnotes: IL, interleukin; MCP, monocyte chemoattractant protein; MIP, macrophage inflammatory protein; IP, Interferon-gamma induced protein; RANTES, regulated on activation IFN, interferon.

**Supplementary Figure S5**

**
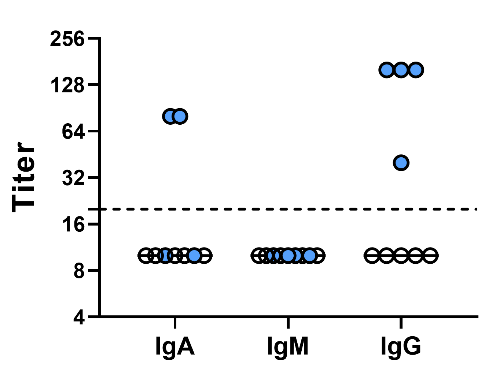
**

**Supplementary Figure 5. Antibody titer detected by immunofluorescence assay (IFA) in household contacts with a positive T-cell response.** IgA, IgM and IgG titers were analyzed. Blue dots indicate subjects with also a concomitant serology response. Antibody titers are expressed as reciprocal of plasma dilution. The dashed line represents the limit of detection of IFA (1:20).

**Supplementary Figure S6**

**
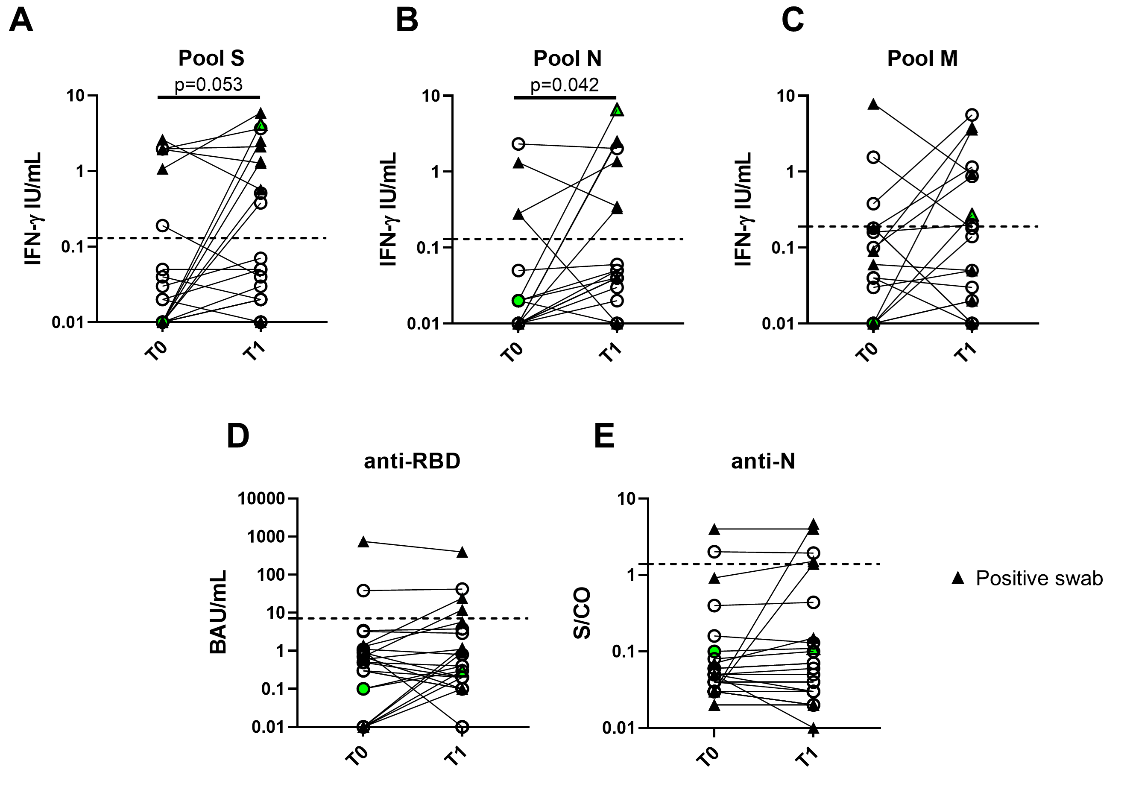
**

**Supplementary Figure 6. Kinetics of T-cell and antibody responses in household contacts of COVID-19 subjects.** Evaluation of the IFN-γ levels (**A**-**C**) and antibody titers (**D**, **E**) in response to SARS-CoV-2 peptides in the household contacts longitudinally sampled at T0 (n=42) and after 7-20 days (T1) (n=28) from the first nasopharyngeal swab. IFN-γ levels were evaluated in plasma harvested from whole-blood samples stimulated with 0.1 µg/mL of pools S (**A**) and M (**C**), and 1 µg/mL of pool N (**B**). Anti-RBD (**D**) and anti-N (**E**) antibodies were evaluated in sera samples and reported as Binding Antibody Units (BAU)/mL and Sample/Cutoff (S/CO), respectively. Black triangles identify subjects with a positive swab as shown in the figure legend. In green is labelled the subject who scored positive only at T1. Dashed lines indicate the cut-off of each test [pools S and N: 0.13 IU/mL; pool M: 0.19 IU/mL; anti-RBD: 7.1 BAU/mL; anti-N: 1.4 (S/CO)]. Footnotes: IFN, interferon; COVID-19, coronavirus disease 19; RBD, receptor-binding-domain; N, nucleoprotein.
